# The Descriptive Data Analysis for the Adoption of Community Cloud in Saudi HEI-Based Factor Adoption

**DOI:** 10.1155/2022/7765204

**Published:** 2022-06-08

**Authors:** Nouf S. Aldahwan, Muhammed S. Ramzan

**Affiliations:** ^1^Department of Information System, King Abdulaziz University, Jeddah, Saudi Arabia; ^2^Department of Information System, King Khaled University, Abha, Saudi Arabia

## Abstract

Due to its increased reliability, adaptability, scalability, availability, and processing capacity, cloud computing is rapidly becoming a popular trend around the world. One of the major issues with cloud computing is making informed decision about adoption of community cloud (CC) computing (ACCC). To date, there are various technology acceptance theories and models to validate perspective of ACCC at both organizational and individual levels. However, no experimental studies have been carried out to provide a comprehensive assessment of the factors of ACCC, specifically in the area of the Saudi Higher Education (HEI) Institution. Thus, this research was aimed at exploring the factors of ACCC and the relationship to the experiences of the employees. The analysis of the employee context was driven by the success factors of technological, organizational, environmental, human, security, and advantage contexts on community cloud computing adoption in HEI. The data collection was a questionnaire-based survey based on 106 responses. We present findings based on descriptive analysis in identifying the significant component that contributed to the effective implementation of ACCC. Security concerns are a significant influencing element in the adoption of community cloud technology.

## 1. Introduction

In the sector of higher education, information and communication technology (lCT) has now become a key player. ICT provides effective cooperation and information sharing platforms, more equity in receiving educational services, and facilitates the gathering and distribution of educational data [1]. Furthermore, using ICT into higher education improves the quality and accuracy of educational services while also lowering costs. Cloud computing has spread swiftly in various educational and business sectors as one of the most rising ICT technologies [2]. Community cloud is no longer a fad, but rather a technology that has the power to change how businesses function. It needs the availability of scalable and flexible computing resources on demand. Individuals and organizations can use the community cloud to access a wide range of essential applications [3]. Many academic institutions are now recognizing the value and relevance of using cloud computing technology in the education sector [4]. For cost savings, organizations with similar criteria and demands use the community cloud. In general, improving system performance requires a community cloud management system among organizations [5]. There are many benefits of community cloud such as reducing cost and workload and increasing the performance and speed [6].

In the field of information technology, community cloud is a major topic. Murugesan and Bojanova [7] defined it as “a deployment model in which a cloud infrastructure is created and deployed in order to be used by a particular community of consumers with shared issues, aims, and interests.” It can be owned and controlled by the community, a third party, or a hybrid of the two. To meet the community's individual requirements and conditions, a network of cloud providers might be able to provide the deploy environment. To establish a global decentralized cloud computing environment, cloud providers can be interconnected via open standards [7].

There are studies that focus on finding the important factor adoption of community cloud as security, privacy, and quality of service (QoS) [8]. In addition, the importance of CC adoption in the context of higher education institutions". And as it becomes clear to the importance of CC computing in reducing costs, as mentioned in the cost-benefit analysis [9]. Community clouds are cloud systems that are adapted to a certain community's needs. CCs have the potential to meet unsatisfied requests of community network members and certain existing services can be made more efficient [10]. Community networks are big, self-organized, and decentralized communication infrastructures that the community builds and operates. They are IP networks that are open, free, and neutral. There are many of community networks which are currently operating around the world, geographically scattered in various parts of the globe without depending on any certain social or economic factors. Individuals, businesses, and organizations all contribute to the infrastructure in a collaborative effort [11].

To assist with this aspect, it is necessary to distribute the survey for the study to get a precise factor. The survey is being used to find out what people think about community cloud adoption as a success element. Data analysis is required to determine appropriate factors for adoption. For the analysis of a community cloud [12], statistical data analysis is applied to examine important data.

During the data analysis, there are a few steps that must be completed. The goal is to ensure that the data is valid and correct. As a result, the initial step is data collection, followed by data analysis. The quantitative technique is used in data analysis [13]. The first section of this paper provides an introduction, while the second section contains the literature review. The research methodology will be present in Section 3. Discussion and data analysis will be explained in Section 4 to meet the research's goals. Section 5 presents the conclusion.

## 2. Literature Review

The article by Aldahwan and Ramzan [14] examines how personnel of IT and telecommunications companies, as well as computer users, feel about community cloud. Examined the current situation of CC adoption in the Kingdom of Saudi Arabia. In addition, presented the motivation factors affecting CC adoption in Saudi Arabia and the problems affecting to CC adoption.

Another work by Heinzlreiter et al. [15], discuss various application situations for a private cloud deployment that can be used as a community cloud for learning and research by various institutions. The research by Valluripally et al. [16] develops a novel community cloud platform to enable clinicians and researchers to have quick access to data sets from diverse sources while preserving data providers' security compliance is not affected. The article by Rodrigues de Castro [17] presents a framework for constructing a community cloud even in scenarios with a high inclusion of nonnative applications. They analyse the architect on a set of infrastructures at High Court of the Brazilian Judicial Branch, which might provide a cost-effective option for other businesses to begin the migration to the cloud model.

Another work by Dubey et al. [18] presents a new management system for serving several enterprises in a safe cloud environment in a community cloud. To coordinate community cloud utilization across organizations, the system uses a virtual server allocation method. The simulation trials show that this method can enhance system capability while meeting deadlines while also lowering monetary costs.

The component cloud service is divided into numerous cooperative communities using the community discovery technique [19]. The community partitioning method may considerably enhance the chances of combined cloud service, according to the testing data. The result shows in increasing the performance level of composite cloud service execution and enhancing the user experience of cloud compound cloud service use.

The article by Aldahwan and Ramzan [20] conducted a comprehensive review of the literature on community cloud adoption and the application of community cloud technology in various industries. The research shows that the community cloud computing technology provides significant benefits to higher education institutions, but that a centralized, well-structured system is required. In addition, they developed a framework to study the factors that influence the adoption of community cloud computing in Saudi HEIs [21].

## 3. Research Methodology

### 3.1. Questionnaire Design

A questionnaire was used to collect data for this study, and a sample from various universities was chosen. The questionnaire has 34 survey items for seven constructs that are like previous literature but have been changed to meet the community cloud computing scenario.

There were two parts to the questionnaire: (a) demographic information and (b) context measurement based on a 5-point Likert scale (agree to strongly disagree), each item of the questionnaire is assessed. The measurement context is based on the following factors: technology readiness, top management support, training, cost, size, complexity, university culture, compatibility, quality of service, mimetic pressures, government support, normative pressures, external support, coercive pressures, usefulness, ease of use, performance, highly automate, adequate resource, cost saving, integrity, governance issue, availability, confidentiality, and privacy.

### 3.2. Study Sampling

Because it is difficult to include or represent all HEls globally in this study, the researcher gives a selection of HEls. The researcher chose the HEls in Saudi Arabia as a study sample. Some universities in Saudi Arabia are among the participants in this investigation.

### 3.3. Data Collection Method

The data is gathered using the questionnaire survey approach for the purpose of evaluating and testing the study model [22]. To begin, specialists in the field of this study evaluate and review the questionnaire to ensure that the survey items are efficient, complete, and relevant. Furthermore, the specialists examine the questionnaire's format, organization, and general layout of the items [23]. Following that, an online version of the questionnaire is created, and a small sample of people from the sample size is randomly chosen to test it. The purpose of this pretest is to confirm that the items are evident and that they are interpreted appropriately, as well as that the format is correct. The pretest findings are then used to make changes to the questionnaire. After then, a link to the questionnaire is given to the intended participants with a cover letter introducing the study's aims and a briefing on cloud computing technology. As a result, all the respondents' responses are received online.

Survey Google Forms was used to create the survey, which received 143 replies. A total of 106 questionnaires were completed after 37 surveys were discarded owing to incorrect or inconsistent responses.

## 4. Data Analysis Discussion

The data analysis is used to interpret and analyse all the information, while the description analysis is used for understanding it. The purpose of descriptive analysis is to find the mean and standard deviation of the data. Using the mean average, the factors with a higher score are indicated as substantial factor adoption.

The success factor needed in community-based adoption was developed using descriptive analysis and the evaluation of the sampling average classification [24]. The analysis mean score and standard deviation score show the result of the employee context that consists of the organization, environment, technology, human, advantages, and security context for the factor adoption.

The following questions aim to measure the support of the adoption of community cloud computing within an organization to get the required data that will be utilized in the quantitative analysis. Question # 1: what are the important organizational factors that influence to the adoption of HEIs in community cloud computing?

Respondents were asked to rate the importance of organization concerns to their organizations. We utilized a one-to-five scale. The five organization elements that received the highest average score were mostly above 3 as shown in Table 1, indicating that organization factors are of considerable concern to most respondents' Technology_readiness (*μ* = 3.62) and the lowest is the size (*μ* = 3.16) are shown in the analysis.

**Figure 1:**
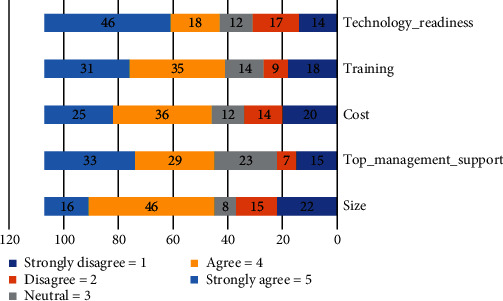
Organization factors to community cloud.

The technology readiness is a major concern as indicated by 46% of respondents as it was rated very important. Of all the respondents, 33% of respondents indicated that the top management support is a very important concern to their organizations. Training was a very important factor for 31% of respondents. Finally, size is a lower concern as indicated by 14% of respondents as it was rated very important as shown in Figure 1.

Question # 2: what are the important technical factors that influence to the adoption of HEIs in community cloud computing? Respondents were asked to rate the importance of technology concerns to their organizations. We utilized a one-to-five scale. The four technology elements that received the highest average score were mostly above 3 as shown in Table 2, indicating that technology factors are of considerable concern to most respondents' University_Culture (*μ* = 3.71) received the best rating, while Quality of Service (*μ* = 2.61) received the lowest.

**Figure 2:**
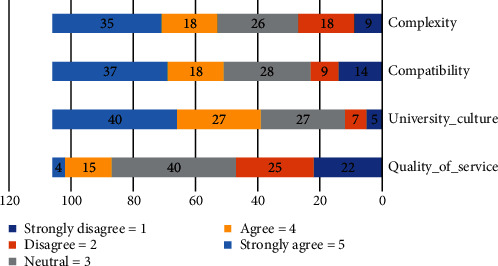
Technology factors to community cloud.

The university culture is a major concern as indicated by 40% of respondents as it was rated very important. Of all the respondents, 37% of respondents indicated that the compatibility is a very important concern to their organizations. Compatibility was a very important factor for 37% of respondents. Finally, quality of service is a lower concern as indicated by 4% of respondents as it was rated very important as shown in Figure 2.

Question # 3. what are the important environmental factors influence to the adoption of HEIs in community cloud computing? Respondents were asked to rate the importance of environment concerns to their organizations. We utilized a one-to-five scale. The environment technical elements that received the highest average score were mostly lower than 3 as shown in Table 3, indicating that environment factors are not more considerable concern to most respondent Mimetic_pressures (*μ* = 3.29), the lowest value is Coercive_pressures (*μ* = 2.65).

**Figure 3:**
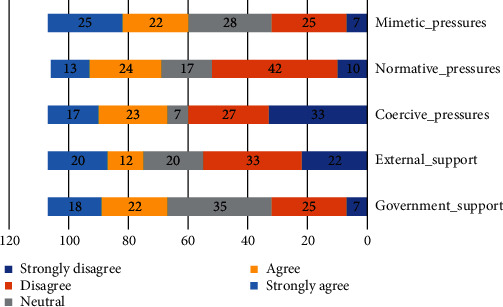
Environment factors to community cloud.

Of all the respondents, 25% of respondents indicated that the mimetic pressures are very important concern to their organizations. External support was a very important factor for 20% of respondents. Finally, normative pressures are a lower concern as indicated by 13% of respondents as it was rated very important as shown in Figure 3.

Question # 4: what are the important human factors that influence the adoption of HEIs in community cloud computing? Table 4 shows respondents were asked to rate the importance of human concerns to their organizations. We utilized a one-to-five scale. The analysis indicates that the highest value is ease of use with *μ* = 3.72, the lowest value is usefulness (*μ* = 2.99).

**Figure 4:**
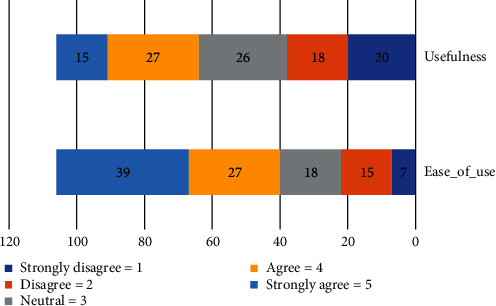
Human factors to community cloud.

The ease of use is a major concern as indicated by 39% of respondents as it was rated very important. Of all the respondents, 15% of respondents indicated that the usefulness is a very important concern to their organizations as shown in Figure 4.

Table 5 shows the mean for an instrument of the advantages context adoption. From the analysis, it shows that the higher mean score is performance (*μ* = 3.26), and the lowest is highly automated (*μ* = 2.76).

**Figure 5:**
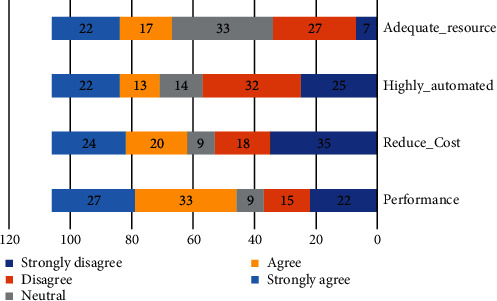
Advantages factors to community cloud.

Question # 5: what are the advantages of community cloud to adoption by Saudi HEIs? Respondents were asked to rate the importance of advantages concerns to their organizations. We utilized a one-to-five scale. Performance was a very important factor for 27% of respondents. High automated and adequate resource indicate 22% of respondents as shown in Figure 5.

Question # 6: what are the important security factors influence that the adoption of HEIs in community cloud computing?Respondents were asked to rate the importance of security concerns to their organizations. We utilized a one-to-five scale. The security elements that received the highest average score were mostly higher than 3 as shown in Table 6, indicating that security factors are more considerable concern to most respondent integrity (*μ* = 3.85), the lowest value is privacy (*μ* = 2.32).

The mean and standard deviation scores for an instrument of security context adoption from the analysis are shown in Table 6.

**Figure 6:**
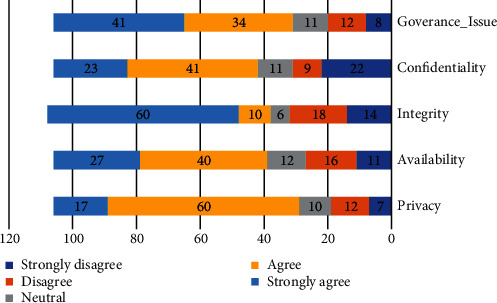
Security factors to community cloud.

The integrity is a major concern as indicated by 60% of respondents as it was rated very important. Of all the respondents, 41% of respondents indicated that the governance issue is a very important concern to their organizations. Finally, privacy is a lower concern as indicated by 17% of respondents as it was rated very important as shown in Figure 6.

**Figure 7:**
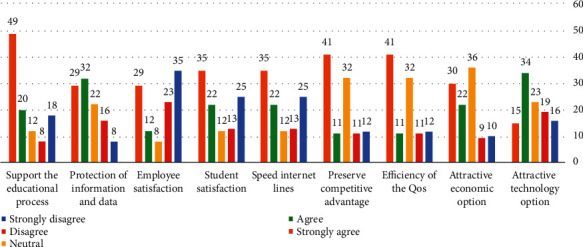
Community cloud adoption.

Question # 7: would you support the adoption of community cloud computing technology in your organization? Respondents were asked to rate the importance of community cloud adoption to their organizations. We utilized a one-to-five scale. The nine community cloud elements that received the highest average score were mostly above 3 as shown in Table 3, indicating that community cloud adoption is a considerable concern to most respondents who support the educational process (*μ* = 3.67) and the lowest is the size (*μ* = 2.77) as shown in the analysis.The support of the educational process is a major concern as indicated by 49% of respondents as it was rated very important. Of all the respondents, 41% indicated that the preservation of the competitive advantage and efficiency of the quality of the services is a very important concern to their organizations. Attractive technology option was a very important factor for 15% of respondents as shown in Figure 7.

Table 7 shows the mean for an instrument of the community cloud computing adoption. From the analysis, it shows that, by higher mean score, the university focuses on modern information technology systems projects that aim to increase the efficiency of the quality of the services and to preserve the competitive advantage (*μ* = 3.52), and the lowest means that the university focuses on modern information technology systems projects that aim to increase employee satisfaction (*μ* = 2.77).

## 5. Conclusion

It is critical to identify the factors (such as data privacy and security and service reliability) that will assist Saudi community service suppliers and technology regulators in developing solutions and strategies that will motivate and enhance the percentage of community cloud adoption for Saudi Universities.

Saudi universities were chosen due to few empirical studies in this field, as well as to assist Saudi universities in their decision-making procedures and subsequent usage of this modern technology, allowing them to gain a competitive edge and keep up with modern technology. This study's contribution is based on the urgent need to identify the advantages, challenges, and other influencing factors.

It is interesting to note that if universities could access scalable technology, they might possibly supply products and services that were previously only available to huge corporations, flattening the competitive landscape.

As a result of the investigation, it has been proved that the security context is vital in assisting the HEI in the adoption of a community-based system. The security ability to provide awareness, motivation, and understanding about the community cloud's benefits is a critical aspect in cloud adoption.

Finally, the data analysis summarizes the analysis of the data gathering step as well as the predicted outcome. These studies go on to present a wide explanation of how to choose the right factors to adopt while moving to the community cloud.

### 5.1. Limitations

Despite its beneficial contributions, the research has some shortcomings. First and foremost, all the participants were IT employees.

Analysing the perspectives of other managers and potential users inside the company could provide greater insight into how user and employee in other departments view community cloud adoption and use. Nonetheless, IT employees were chosen as respondents since they needed to have a better understanding of community cloud technologies and services. The sample size is also a constraint.

### 5.2. Suggestions for Future Work

Supplemental research is needed to investigate the proposed hypotheses in a variety of contexts, as well as to assess the reliability of the measures used to help organizations better understand the success obstacles of community cloud adoption in other growth of emerging sectors of the economy. Future studies should also aim at increasing the sample size and diversity of respondents.

Collecting data from people other than IT employee and specialists, for example, can help you understand how people feel about the adoption of this technology from a variety of viewpoints and interests.

Additional factors, such as employee training, business culture, and the conditions of the cloud service provider's agreement, can provide more insight into what really impacts technology adoption. Community cloud is pervading practically every aspect of our lives.

## Figures and Tables

**Table 1:** Organization mean score.

Organization	Mean score	Standard deviation
Technology_readiness	3.62	1.48
Top_management_support	3.53	1.36
Cost	3.31	1.44
Training	3.47	1.42
Size	3.16	1.40

**Table 2:** Technology mean score.

Technology	Mean score	Standard deviation
Complexitiy	3.61	1.37
Compatibility	3.50	1.40
University_Culture	3.71	1.35
Quality_of_Service	2.61	1.10

**Table 3:** Environment mean score.

Environment	Mean score	Standard deviation
Goverment_support	3.23	1.11
External_support	2.76	1.39
Coercive_pressures	2.65	1.49
Normative_pressures	2.89	1.22
Mimetic_pressures	3.29	1.24

**Table 4:** Human mean score.

Human	Mean score	Standard deviation
Usefulness	2.99	1.32
Ease_of_use	3.72	1.27

**Table 5:** Advantages mean score.

Advantages	Mean score	Standard deviation
Performance	3.26	1.50
Reduce_Cost	2.81	1.60
Highly_automated	2.76	1.47
Adequate_resource	3.19	1.22

**Table 6:** Security mean score.

Security	Mean score	Standard deviation
Privacy	3.32	1.44
Availability	3.64	1.08
Integrity	3.85	1.50
Confidentiality	3.53	1.30
Goverance_Issue	3.82	1.26

**Table 7:** Community cloud computing mean score.

Advantages	Mean score	Standard deviation
Community cloud technology is an attractive technology option for the university.	3.066	1.28188
Community cloud technology is an attractive economic option for the university.	3.4717	1.22052
The university focuses on modern information technology systems projects that aim to increase the efficiency of the quality of the services it provides to the beneficiaries.	3.5283	1.38156
The university focuses on modern information technology systems projects that aim to preserve the competitive advantage.	3.5283	1.38156
The university has high-speed Internet lines, and its services are uninterrupted.	3.2547	1.58602
The university focuses on modern information technology systems projects that aim to increase student satisfaction.	3.2547	1.58602
The university focuses on modern information technology systems projects that aim to increase employee satisfaction.	2.7736	1.64625
The university focuses on modern information technology systems projects that aim to increase the protection of information and data.	3.5283	1.24371

## Data Availability

The SPSS data used to support the findings of this study have not been made available for privacy reason.
